# Diffusion characteristics classification framework for identification of diffusion source in complex networks

**DOI:** 10.1371/journal.pone.0285563

**Published:** 2023-05-15

**Authors:** Fan Yang, Jingxian Liu, Ruisheng Zhang, Yabing Yao

**Affiliations:** 1 Key Laboratory of Intelligent Information Processing and Graph Processing, Guangxi University of Science and Technology, Liuzhou, Guangxi, China; 2 School of Computer Science and Technology, Guangxi University of Science and Technology, Liuzhou, Guangxi, China; 3 School of Information Science and Engineering, Lanzhou University, Lanzhou, Gansu, China; 4 School of Computer and Communication, Lanzhou University of Technology, Lanzhou, Gansu, China; University of Parma, ITALY

## Abstract

The diffusion phenomena taking place in complex networks are usually modelled as diffusion process, such as the diffusion of diseases, rumors and viruses. Identification of diffusion source is crucial for developing strategies to control these harmful diffusion processes. At present, accurately identifying the diffusion source is still an opening challenge. In this paper, we define a kind of diffusion characteristics that is composed of the diffusion direction and time information of observers, and propose a neural networks based diffusion characteristics classification framework (NN-DCCF) to identify the source. The NN-DCCF contains three stages. First, the diffusion characteristics are utilized to construct network snapshot feature. Then, a graph LSTM auto-encoder is proposed to convert the network snapshot feature into low-dimension representation vectors. Further, a source classification neural network is proposed to identify the diffusion source by classifying the representation vectors. With NN-DCCF, the identification of diffusion source is converted into a classification problem. Experiments are performed on a series of synthetic and real networks. The results show that the NN-DCCF is feasible and effective in accurately identifying the diffusion source.

## Introduction

Most complex systems in the real world take the form of networks [1] in which the nodes and edges denote the units and the interactions between units, respectively. Various diffusion phenomena taking place in networks are usually modelled as diffusion process [1], such as disease spreading [2], rumor diffusion [3] and computer virus propagation [4]. The ubiquity of these harmful diffusion processes has incurred huge losses to human society. Therefore, it is of great theoretical and practical significance to develop effective strategies to control the harmful diffusion process. One of the important measures is identifying the diffusion source that initiates the diffusion process on networks, which has attracted widespread attentions in recent years [5]. Many existing source identification methods provided effective solutions for some important issues in reality, such as identifying the source of SARS [6], COVID-19 [7], Cholera [8], finding the source of foodborne disease [9], etc. However, accurately identifying the diffusion source is still an opening challenge.

The success of artificial neural networks has boosted research on many scientific fields [10–12]. Especially, the emergence of graph neural networks [13, 14] (GNNs) and network embedding [15, 16] facilitate the applications of artificial neural networks on irregular structures of networks. GNNs are the neural network models to address different graph tasks in an end-to-end way [13]. The most common GNNs include recurrent graphs neural networks [17], convolutional graph neural networks [18], graph autoencoders [13], etc. Network embedding is composed of various kinds of methods designed for a same task, i.e., network representation learning [13]. Recently, GNNs and network embedding have been successfully introduced into some important issues of complex networks [13, 16], such as link prediction and node classification. However, only a few artificial neural networks based methods focused on the diffusion source identification problem [19, 20]. Li et al. [19] proposed a label propagation framework to locate the diffusion source. Due to the common characteristics between label propagation framework and graph convolutional networks (GCNs), the source identification is converted into a multi-classification problem. Dong et al. [20] detected multiple sources by utilizing the wavefront information. Since existing GNNs is not a suitable solution for the wavefront based method, they developed a novel multi-task learning model based on encoder-decoder structure. Different from the two methods in [19] and [20], this paper utilizes the diffusion time and direction information recorded in limited observers to identify the diffusion source. The two types of information have been proved to be helpful in accurately identifying the source [21–26]. We define the two types of information as diffusion characteristics, and identify the diffusion source by classifying the diffusion characteristics. Although existing GNNs and network embedding are powerful models to process graph data, both of them are not suitable to be used to process the diffusion characteristics which is dynamically generated in a diffusion process. Therefore, we develop a novel neural networks based diffusion characteristics classification framework, which contains the following three stages, (*i*) the diffusion characteristics are utilized to construct network snapshot feature, (*ii*) a graph LSTM auto-encoder is proposed, by which the network snapshot feature is represented as low-dimension vectors, (*iii*) a source classification neural network is proposed to identify the diffusion source by classifying the representation vectors of network snapshot feature. With the proposed framework, the identification of diffusion source is converted into a classification problem. Further, the feasibility and effectiveness of this framework is validated by the experimental results.

The rest of this paper is organized as follows. Existing related works are briefly reviewed in Section Related work. The neural networks based diffusion characteristics classification framework is proposed in Section Materials and methods. The experimental results are discussed in Section Results. We conclude this work in Section Conclusion.

## Related work

The early diffusion source identification methods were developed for unweighted networks. A systematic method was pioneered by Shah et al. [27], they constructed a source estimator based on a topological quantity termed as Rumor Centrality (RC). The RC has been extended to identify the diffusion source in more complex environments [28–30]. Zhu et al. [31] proposed a sample path based method termed as Jordan Center (JC), which has been improved to identify the diffusion source with limited observations [32–34]. Meanwhile, many methods based on various ideas were proposed for unweighted networks, including the Dynamic Message Passing based method [35], the Belief Propagation based method [36], the Monte-Carlo method based method [37], the Rationality Observation based method [38], the Label Ranking framework based method [39], the Time Aggregated Graph based method [40], etc. The above methods are effective in unweighted networks. However, in reality, we have to consider various significant weights associated with the edges in networks, such as the traffic, the time delay and so on.

For weighted networks, Brockmann et al. [6] modeled the Global Mobility Network as a weighted graph, and identified the epidemic source based on a novel effective distance. This method has been extended to identify multi-source by Jiang et al. [41]. Meanwhile, several methods based on various ideas were proposed to identify the diffusion source in weighted networks [42–44]. However, these methods require the knowledge of all nodes state. In reality, it is often the case that only limited nodes state can be observed [45]. For this problem, many methods were proposed by utilizing limited observers, including the Time-Reversal Backward Spreading algorithm [24], the Backward Diffusion-based method [46], the improved Gaussian estimator [47], the Gromov matrix based method [25], the Greedy Optimization based algorithm [26], the Sequential Neighbour Filtering algorithm [48], the Estimated Propagation Delay based algorithms [49], etc. These methods [24–26, 46–49] mainly utilized the diffusion time information of observers to identify the source. Pinto et al. [50] proposed a Gaussian estimator, which is the first method to identify the source by utilizing the diffusion direction information of observers. However, the diffusion direction information is only used in the tree graphs. Yang et al. [21] improved the accuracy of Gaussian estimator on general graphs by utilizing the diffusion direction information of observers. Zhu et al. [22, 23] also proposed a path-based source identification method by utilizing the diffusion direction information of observers. Obviously, the diffusion time and direction information of observers play important roles in accurately identifying the diffusion source.

Different from all the traditional source identification methods mentioned above, in recent years, a few artificial neural networks based methods are developed to identify the source. Li et al. [19] proposed a Source Identification Graph Convolutional Network (SIGN) framework, this method requires the knowledge of complete observation. Dong et al. [20] proposed a graph constraint based sequential source identification model. To obtain the wavefront information, this method [20] also requires the knowledge of complete observation. However, in reality, it is often the case that only limited nodes state can be observed [45]. In this paper, we identify the diffusion source by utilizing limited observers. We define the diffusion time and direction information of observers as diffusion characteristics, and propose an artificial neural networks based framework to identify the source by classifying the diffusion characteristics. The feasibility and effectiveness of the proposed framework are validated on a series of synthetic and real networks.

## Materials and methods

### Problem description and overview

A network the diffusion process taking place in is modelled as a finite and undirected graph G={V,E,θ}, where *V* and *E* represent the nodes set and edges set, respectively. ***θ*** = {*θ*_*vu*_}, *θ*_*vu*_ is the random propagation delay associated with an edge *vu*, *vu* ∈ *E*. Generally, G is assumed to be known. We consider that the {*θ*_*vu*_} associated with *E* are independent and identically distribution (I.I.D) random variables.

#### Diffusion model

Assuming that the diffusion process taking place in G follows a simple diffusion model that is similar to reference [50]. At time *t*, each node *v* ∈ *V* is only in one of the two states: (*i*) informed, if it has received the information from any one neighbour, or (*ii*) ignorant, if it has not been informed so far. Any node *v* is equally likely to be the source. The diffusion process is initiated by a single source s* at unknown start time, all nodes are ignorant except for s* is informed. Let N(v) denote the neighbour(s) of *v*. Suppose *v* is in the ignorant state, and receives the information for the first time from one informed neighbour *w*, thus becoming informed at time *t*_*v*_. Then, *v* will attempt to retransmit the information to all its other neighbours along the edges, so that each neighbour u∈N(v)\w receives the information with success probability *β* at time *t*_*v*_ + *θ*_*vu*_. If there are two or more informed neighbours having a same propagation delay to *u*, *u* can be informed by only one neighbour. Once the diffusion process is terminated, a network snapshot, denoted by S, will be generated.

For an arbitrary G={V,E,θ}, with the diffusion model introduced above, a network snapshot S is generated. Generally, only a part of nodes state in S can be observed, we call these nodes observers, denoted by O. The observations made by O provide two types of information [21, 50]: (*i*) the direction in which information arrives to observers and, (*ii*) the timing at which the information arrives to observers. Obviously, the two types of information recorded in O show the true details of the diffusion process, which have been proved to be helpful in accurately identifying the diffusion source [21–26]. In this paper, the two types of information are defined as diffusion characteristics. The purpose is to find the diffusion source s* from S by utilizing the diffusion characteristics recorded in O. We propose a neural networks based diffusion characteristics classification framework (NN-DCCF) to identify the diffusion source, by which the identification of source is converted into a classification problem. NN-DCCF is composed of the following three stages.

1. By selecting vital nodes and extending their neighbours in a given G, we build O. Then, for a S, by utilizing the diffusion characteristics recorded in O, we construct network snapshot feature, denoted by F(S).
2. We propose a graph LSTM auto-encoder (GLSTM-AE). By using GLSTM-AE, F(S) is represented as low-dimension vectors, denoted by R(S).
3. We propose a source classification neural network (SCNN) to estimate the diffusion source by classifying R(S).

**Fig 1:**
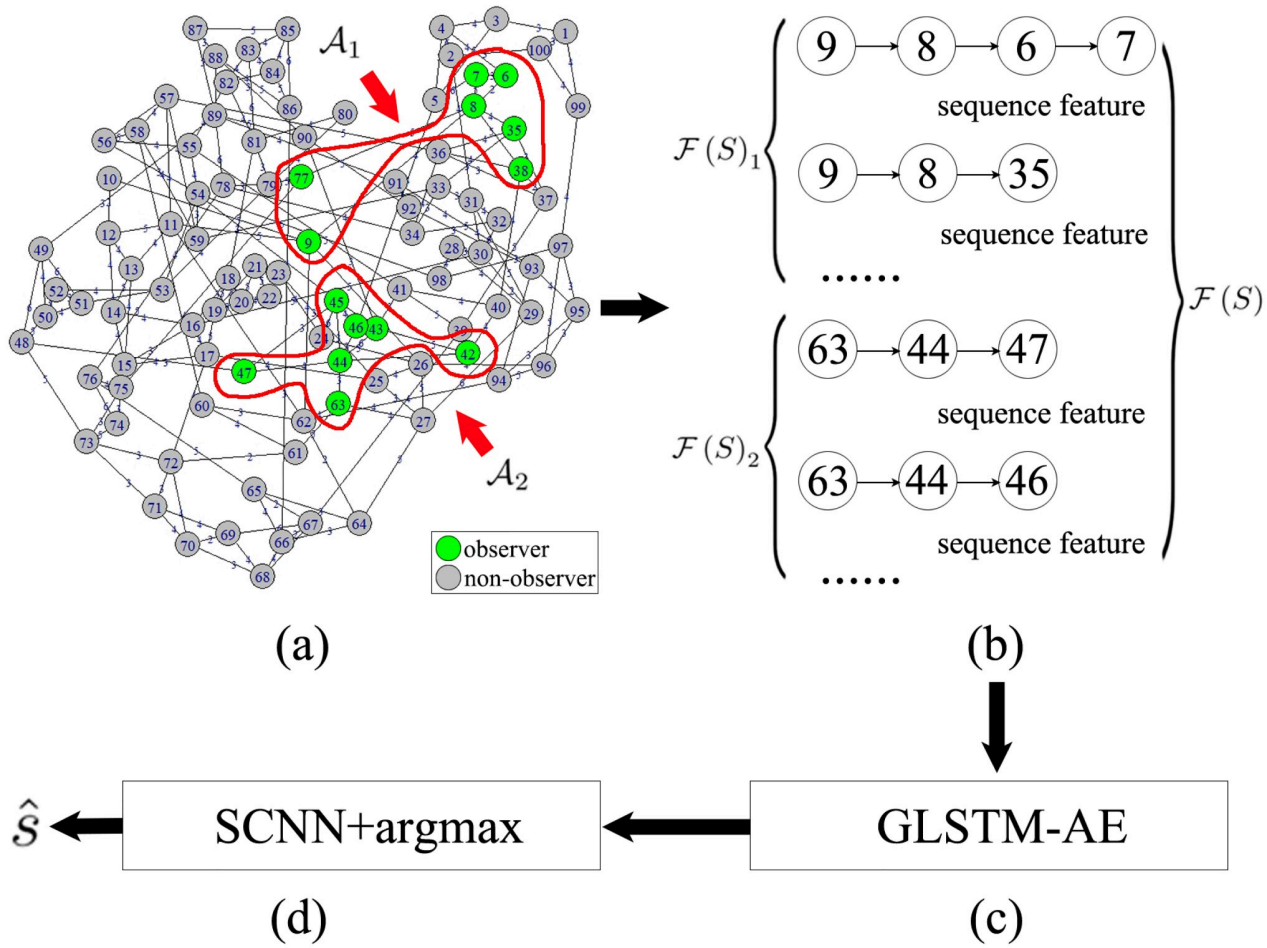
Overview of NN-DCCF. (a) The vital nodes selected by degree centrality [51] include node 8 and node 44. Observation areas set $A=\{A_{1},A_{2}\}$. $A_{1}=\{6,7,8,9,35,38,77\}$. $A_{1}$ consists of node 8 and its neighbours within 1 hop distance. $A_{2}=\{42,43,44,45,46,47,63\}$. $A_{2}$ consists of node 44 and its neighbours within 1 hop distance. $O=\{A_{1}\cup A_{2}\}$. (b) $F(S)=\{F{\left(S\right)}_{1},F{\left(S\right)}_{2}\}$. $F{\left(S\right)}_{1}$ is composed of the sequence features constructed with the diffusion characteristics recorded in the observers of $A_{1}$. $F{\left(S\right)}_{2}$ is composed of the sequence features constructed with the diffusion characteristics recorded in the observers of $A_{2}$. (c) By using GLSTM-AE, F(S) is converted into low-dimension representation vectors, i.e. R(S). (d) With R(S) as the input of SCNN, we can estimate the diffusion source.

The overview of NN-DCCF is shown in Fig 1. Frequently used notations are summarized in Table 1.

**Table 1:** Notation summarization.

Notation	Definition
G	the topological graph of network
*V*	the nodes set in G
*E*	the edges set in G
*o*	observer
O	observers set
***θ***	propagation delay set associated with *E*
S	network snapshot
s*	diffusion source
*β*	propagation rate of diffusion model
*K*	the number of vital nodes
A	observation areas set
F(S)	network snapshot feature
R(S)	the representation vectors of F(S)
*l*	the length of sequence feature
*l*_max_	the max length of sequence feature
*η*	the number of sequence features
|⋅|	the number of elements

### Stage 1: Constructing network snapshot feature

To utilize the diffusion characteristics of observers to construct network snapshot feature, the observers set O is built with the following strategy. Given a G, we first rank the importance of nodes by a vital nodes identification methods [51]. Next, with the ranking results, we select the most important *K* nodes as vital nodes. Then, for each vital node, we extend its neighbours within *h* hops distance. Further, each vital node and its extended neighbours are combined to form an observation area. G contains *K* observation areas $A_{1},A_{2},\cdots ,A_{K}$, $A={\left\{A_{i}\right\}}_{i=1}^{K}$, $O=\{A_{1}\cup A_{2}\cup \cdots \cup A_{K}\}$. *o* denotes an unique observer in O. When the diffusion process occurs on G and generates S, by utilizing the diffusion characteristics, i.e., the diffusion direction and time information, recorded in each o∈O, we construct the network snapshot feature F(S). Here, we set K=|A|=|F(S)|. The procedure for constructing F(S) is summarised in Algorithm 1.

**Algorithm 1** Network snapshot feature constructing algorithm

**Input:** G , A and S

**Output:** F(S)

1: initialize an empty $F(S)={\left\{F{\left(S\right)}_{i}\right\}}_{i=1}^{\left|A\right|}$

2: sort all $A_{i}$ in A according to the average informed time of $A_{i}$

3: **for** each $A_{i}$ in A **do**

4:  **for** each $o\in A_{i}$ **do**

5:   initialize an empty *seq* to record a single sequence feature

6:   set current node *c* = *o*

7:   **while** $c\in A_{i}$ **do**

8:    **if** *c* is in the informed state **then**

9:     add *c* into *seq*

10:     get next node *n* according to the diffusion direction information recorded in *c* and set *c* = *n*

11:    **end if**

12:   **end while**

13:   reverse the nodes order in *seq*

14:   **if** 1 < |*seq*| ≤ *l*_max_ **then**

15:    add *seq* into $F{\left(S\right)}_{i}$

16:   **else if** |*seq*| > *l*_max_ **then**

17:    remove the last |*seq*| − *l*_max_ nodes from *seq*

18:    add *seq* into $F{\left(S\right)}_{i}$

19:   **end if**

20:  **end for**

21: **end for**

22: **for** each $F{\left(S\right)}_{i}$ in F(S) **do**

23:  remove duplicated sequence features from $F{\left(S\right)}_{i}$

24:  sort all sequence features in $F{\left(S\right)}_{i}$ according to their length

25:  **if** $|F{\left(S\right)}_{i}|>\eta$ **then**

26:   remove the last $|F{\left(S\right)}_{i}|-\eta$ sequence features from $F{\left(S\right)}_{i}$

27:  **end if**

28: **end for**

In Algorithm 1, the inputs are the topology of G, observation areas set A and network snapshot S. The average informed time of $A_{i}$ in step 2 is the average of the diffusion time information recorded in $o\in A_{i}$. Steps 4–20 are used for constructing the sequence features in $F{\left(S\right)}_{i}$ by traversing each $o\in A_{i}$. Here, steps 7–12 are used to generate a single sequence feature, denoted by *seq*. A single *seq* is a basic unit for constructing F(S). Obviously, generating a single *seq* depends on the diffusion direction information of observers. Step 13 is used to reverse the order of current *seq*. Steps 14–19 are used to add the *seq* into $F{\left(S\right)}_{i}$, where, 2 ≤ |*seq*| ≤ *l*_max_. Further, from step 3 to step 21, the sequence features in each $F{\left(S\right)}_{i}$ are constructed, then, we get F(S). Steps 22–28 are used to remove the redundant sequence features and limit the size of F(S). A schematic to obtain F(S) by using Algorithm 1 is shown in Fig 1(a) and 1(b).

### Stage 2: GLSTM-AE based network snapshot feature representation

**Fig 2:**
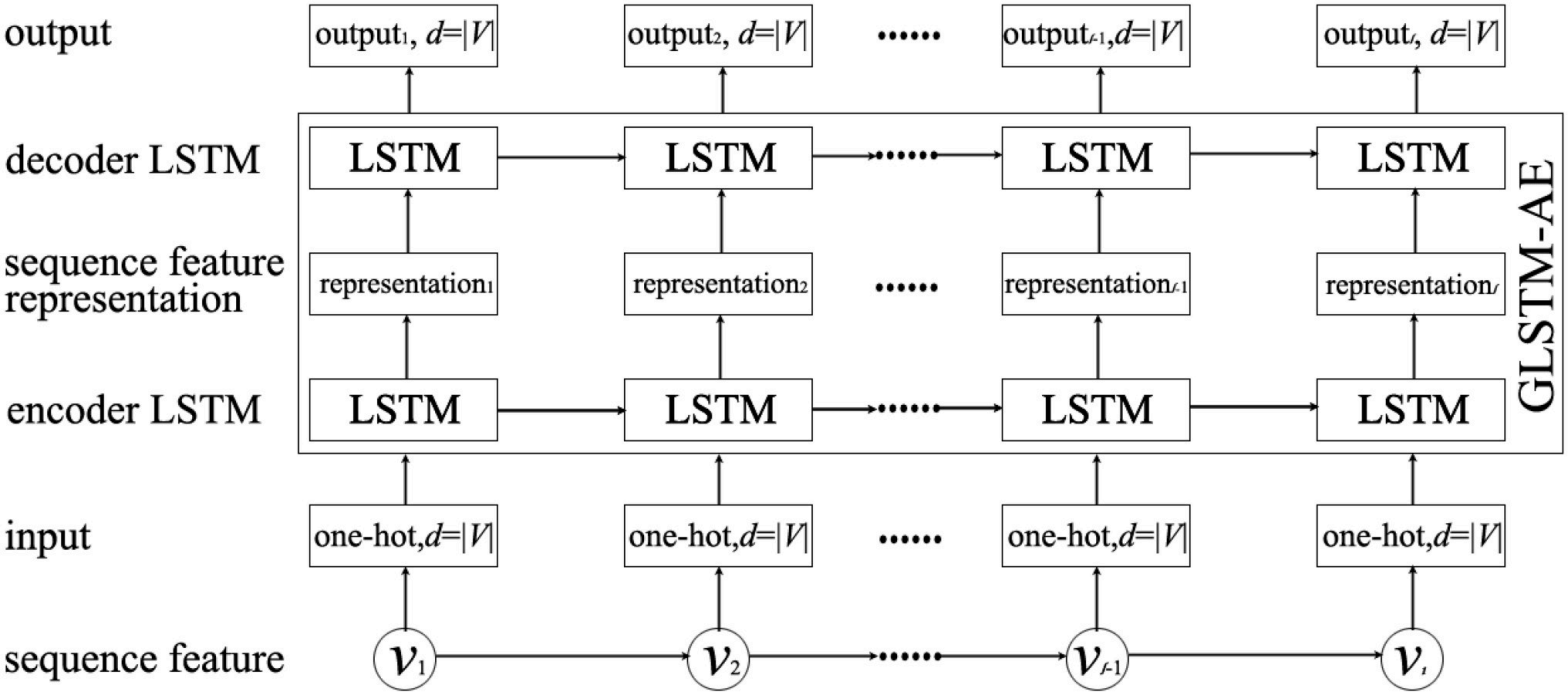
The structure of GLSTM-AE, where, *d* denotes the dimension of a vector.

From Algorithm 1, we know that each sequence feature, termed as *seq*, in F(S) consists of several ordered informed nodes. Therefore, the *seq* is a type of sequential data. Inspired by the idea that the long short-term memory (S1 File) is a powerful tools for modelling sequential data [52–54], we use the LSTM networks to learn the representation of *seq*. However, the *seq* is different from traditional sequential data since it is composed of ordered informed nodes. Further, we propose a graph LSTM auto-encoder (GLSTM-AE) to learn the low-dimension representation of *seq*. A GLSTM-AE consists of two LSTMs, the encoder LSTM and the decoder LSTM, as shown in Fig 2. GLSTM-AE works as follows. For an arbitrary *seq*, each node in *seq* is represented as an one-hot vector with dimension |*V*|. The input to GLSTM-AE is the one-hot representation of *seq*. The output of the encoder LSTM after the last input has been read is low-dimension representations of the one-hot vectors of *seq*, denoted by ***r***, $r\in R^{\left|seq\right|\times d_{r}}$, where, *d*_*r*_ denotes the representation dimension. ***r*** is the representation result we obtained from the GLSTM-AE. The decoder LSTM reconstruct back the input from ***r***. The target of GLSTM-AE is same as the input.

Obviously, it is necessary to train GLSTM-AE before it is applied to learn the representations of the sequence features in F(S). A simple way to obtain the training data of GLSTM-AE is generating sequence features with fixed length from G.

Because the mean squared error (MSE) loss is commonly used for the regression task, it is suitable for the task of GLSTM-AE. Therefore, we adopt the MSE loss as the loss function of GLSTM-AE, which is described as follows.

$$\begin{array}{c} Loss_{GLSTM-AE}=MSELoss(Y,Y^{*}) \end{array}$$ (1)

where, *Y* denotes the output of the decoder LSTM in GLSTM-AE, Y* denotes the one-hot representation of *seq*.

Then, with the trained GLSTM-AE, we get the low-dimension representation of F(S), denoted by R(S). This process is summarised in Algorithm 2.

Algorithm 2 Network snapshot feature representation algorithm

**Input:** F(S)

**Output:** R(S)

1: initialize an empty R(S), $R(S)\in R^{K\times \eta \times l_{\text{max}}\times d_{r}}$

2: set $p_{l}=[0]\in R^{d_{r}}$

3: set $p_{\eta }=[0]\in R^{l_{\text{max}}\times d_{r}}$

4: **for** each $F{\left(S\right)}_{i}$ in F(S) **do**

5:  **for** each *seq* in $F{\left(S\right)}_{i}$ **do**

6:   ***input***=one-hot(*seq*), ***input*** ∈ **R**^|*seq*|×|*V*|^

7:   ***r*** = GLSTM-AE (***input***), $r\in R^{\left|seq\right|\times d_{r}}$

8:   **if** |*seq*| < *l*_max_ **then**

9:    *k* = *l*_max_ − |*seq*|

10:    pad ***r*** with ***p***_*l*_ for *k* times

11:   **end if**

12:   add ***r*** into $R{\left(S\right)}_{i}$

13:  **end for**

14:  **if** $|F{\left(S\right)}_{i}|<\eta$ **then**

15:   $k=\eta -|F{\left(S\right)}_{i}|$

16:   pad $R_{i}(S)$ with ***p***_*η*_ for *k* times

17:  **end if**

18: **end for**

In Algorithm 2, the input is the network snapshot feature F(S). The one-hot(⋅) function in step 6 is to get the one-hot representation of current *seq*. In step 7, the representation result ***r*** of *seq* is obtained by using the trained GLSTM-AE. Further, we set $r\in R^{l_{\text{max}}\times d_{r}}$ by steps 8–11, and set $R_{i}\in R^{\eta \times l_{\text{max}}\times d_{r}}$ by steps 14–17.

### Stage 3: Identify the diffusion source with SCNN

**Fig 3:**
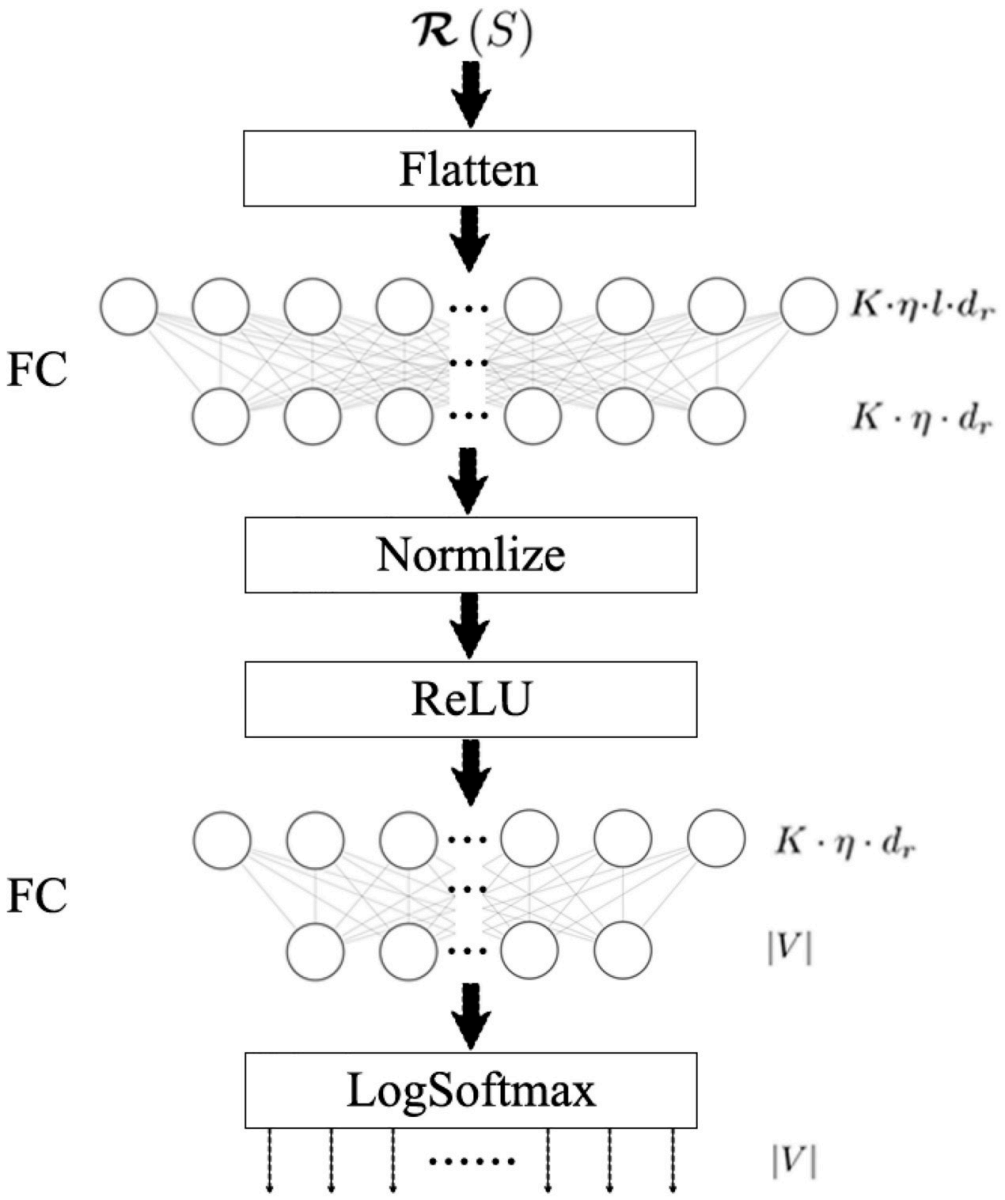
The structure of SCNN.

With Algorithm 2, we get the representation of F(S), i.e. R(S). In this section, with R(S) as input, we propose a source classification neural network (SCNN) to identify the diffusion source by classifying R(S). SCNN is mainly composed of two fully connected layers. To get convergence faster, we add a normalization layer. The structure of SCNN is shown in Fig 3, where, the LogSoftmax is used for multi-class classification.

SCNN also requires to be trained before it is applied to identify the diffusion source. The training data of SCNN can be generated by Algorithm 3.

**Algorithm 3** SCNN training data generating algorithm

**Input:** G and A

**Output:** training data collector *C*

1: specify the number of loops *N*

2: initialize an empty training data collector *C*

3: set $\beta ={\left\{\beta_{i}\right\}}_{i=1}^{M}$, *β*_*i*_ ∈ (0, 1), ∀*i*, *j* ∈ [1, *M*], *β*_*i*_ ≠ *β*_*j*_

4: **while** *N* > 0 **do**

5:  **for** *β*_*i*_ ∈ ***β* do**

6:   **for** each node *v* ∈ *V* **do**

7:    generate S by running the diffusion model (see Diffusion model) on G with *v* as diffusion source and *β*_*i*_ as propagation rate

8:    generate F(S) by Algorithm 1

9:    construct R(S) corresponding to F(S) by Algorithm 2

10:    add a training data (R(S), one-hot(*v*)) into *C*

11:   **end for**

12:  **end for**

13:  *N* = *N* − 1

14: **end while**

In Algorithm 3, the inputs are the topology of G and observation areas set A. From step 7 to step 10, given a node *v* and a propagation rate *β*, a single training data can be generated, which is composed of the R(S) and the one-hot representation of *v*. Obviously, the SCNN training dataset size is *N* ⋅ |*β*| ⋅ |*V*|.

Because the cross entropy loss is mainly used for classification, we adopt the cross entropy loss as the loss function of SCNN, which is described as follows.

$$\begin{array}{c} Loss_{SCNN}=CrossEntropyLoss(Z,Z^{*}) \end{array}$$ (2)

where, *Z* denotes the estimated diffusion source obtained by SCNN, Z* denotes the one-hot representation of true diffusion source.

Finally, by combining with the trained SCNN, the algorithm corresponding to NN-DCCF is summarised as Algorithm 4.

**Algorithm 4** Diffusion source identification algorithm

**Input:** G , A and S

**Output:** $\hat{s}$

 1: generate F(S) according Algorithm 1

 2: construct R(S) corresponding to F(S) according to Algorithm 2

 3: ***output*** = SCNN (R(S)), ***output*** ∈ **R**^|*V*|^

 4: $\hat{s}=\text{arg}\;\text{max}(\text{output})$

## Results

### Main experimental environment

Hardware: Dell R740 with 2 Intel(R) Xeon(R) gold 6254 CPU, 1 TB RAM, 1 NVIDIA Tesla V100S GPU with 32 GB GPU memory. Software: Python 3.8.10 + PyTorch 1.10.2 + CUDA 10.2.

### Methods for comparison

Essentially, the proposed NN-DCCF is an observers based method. To validate its feasibility and effectiveness, three existing state-of-the-art observers based methods are selected for comparison, including time-reversal backward spreading (TRBS) algorithm [24], sequential neighbour filtering (SNF) algorithm [48] and estimated propagation delay (EPD) algorithm [49].

### Datasets

We compare the four diffusion source identification methods on a series of synthetic and real networks. The synthetic networks include scale-free (BA) [55] model and small-world (WS) [56] model. The parameters for generating synthetic networks are summarised in Tables 2 and 3. The real networks are of different types, including NetworkScience (https://networkrepository.com/ca-netscience.php) [57], Euroroads (https://networkrepository.com/subelj-euroroad.php) [57], Email (https://networkrepository.com/email-univ.php) [57] and Blogs (https://doi.org/10.1007/978–3-642-01206-8_5) [58]. The topological properties of all networks are summarised in Table 4.

**Table 2:** The parameters for generating BA models.

Network	|*V*|	*M*	*power*
BA model (1)	400	2	1.3
BA model (2)	1000	2	1.3

*M*: the number of outgoing edges generated for each node.

*power*: the power of the preferential attachment [55].

**Table 3:** The parameters for generating WS models.

Network	|*V*|	*Nei*	*p*
WS model (1)	400	2	0.1
WS model (2)	1000	2	0.1

*Nei*: the size of the neighbourhood for each node.

*p*: the rewiring probability [56].

**Table 4:** The topological properties of networks.

Network	|*V*|	|*E*|	〈*k*〉	*A*	*H*	*APL*
BA model (1)	400	797	3.99	−0.166	2.64	3.55
BA model (2)	1000	1997	3.99	−0.131	8.45	3.41
WS model (1)	400	800	4.00	−0.014	1.05	5.87
WS model (2)	1000	2000	4.00	0.038	1.05	6.85
NetworkScience	379	914	4.82	−0.082	1.66	6.04
Euroroads (LCC)	1039	1305	2.51	0.090	1.23	18.4
Email	1133	5451	9.62	0.078	1.94	3.61
Blogs	3982	6803	3.42	−0.133	4.04	6.25

〈*k*〉: average degree.

*A*: the assortative coefficient [59].

*H*: the degree heterogeneity [60]

$H=\frac{\langle k^{2}\rangle }{{\langle k\rangle }^{2}}$ .

*APL*: the average path length (the number of edges).

LCC: largest connected component.

### Evaluation metrics

The performance of diffusion source identification methods are commonly evaluated with two metrics [5, 21, 25, 27, 34], including the precision and average error distance. The precision focuses on evaluating the capability of a method in precise identification (i.e. the proportion of 0 error hop). For each network, we randomly select 100 nodes as test seeds. For the precision, the higher the value is, the better the algorithm is. For the average error hop, the smaller the value is, the better the algorithm is.

### Parameters setting

For an arbitrary G={V,E,θ}, we assume that ***θ*** are Gaussian distribution $N(\mu ,\sigma^{2})$ [24, 49], *μ* and *σ*^2^ are known [50], here, we set *μ*/*σ* = 4 [21, 50]. We assume that the diffusion process on networks follows the diffusion model introduced in Diffusion model. To investigate the performance of NN-DCCF under different propagation rates, we set relatively larger range for *β*, *β* ∈ [0.1, 0.9]. The diffusion process is terminated when there is no ignorant node.

How to select a suitable observers placement strategy may depends on the topology of a network [61]. Although lots of methods [51] can be used to select O, sometimes there maybe no significant difference between the placement strategies for the performance of source identification [62]. In this paper, O is selected by the strategy introduced in Section Stage 1. Here, the *K* vital nodes in G are selected by the degree centrality (DC) [51] (due to the simplicity and efficiency of DC). For each network, we select 1% nodes as vital nodes. Then, by extending the neighbours within 1 hop distance of the vital nodes, we get A and O in G, the details are shown in Table 5. Other general parameters are also summarised in Table 5. All the four compared methods adopt the same O to identify the diffusion source.

**Table 5:** General parameters setting.

Network	|*V*|	*K*	|A|	|O|	*l*_max_	*η*
BA model (1)	400	4	4	144	4	8
BA model (2)	1000	10	10	594	4	8
WS model (1)	400	4	4	32	4	8
WS model (2)	1000	10	10	76	4	8
NetworkScience	379	4	4	73	4	10
Euroroads	1039	11	11	88	4	6
Email	1133	12	12	355	4	20
Blogs	3982	40	40	1646	4	8

*η* = 2 ⋅ ⌈〈*k*〉⌉, 〈*k*〉 can be found in Table 4.

**Fig 4:**
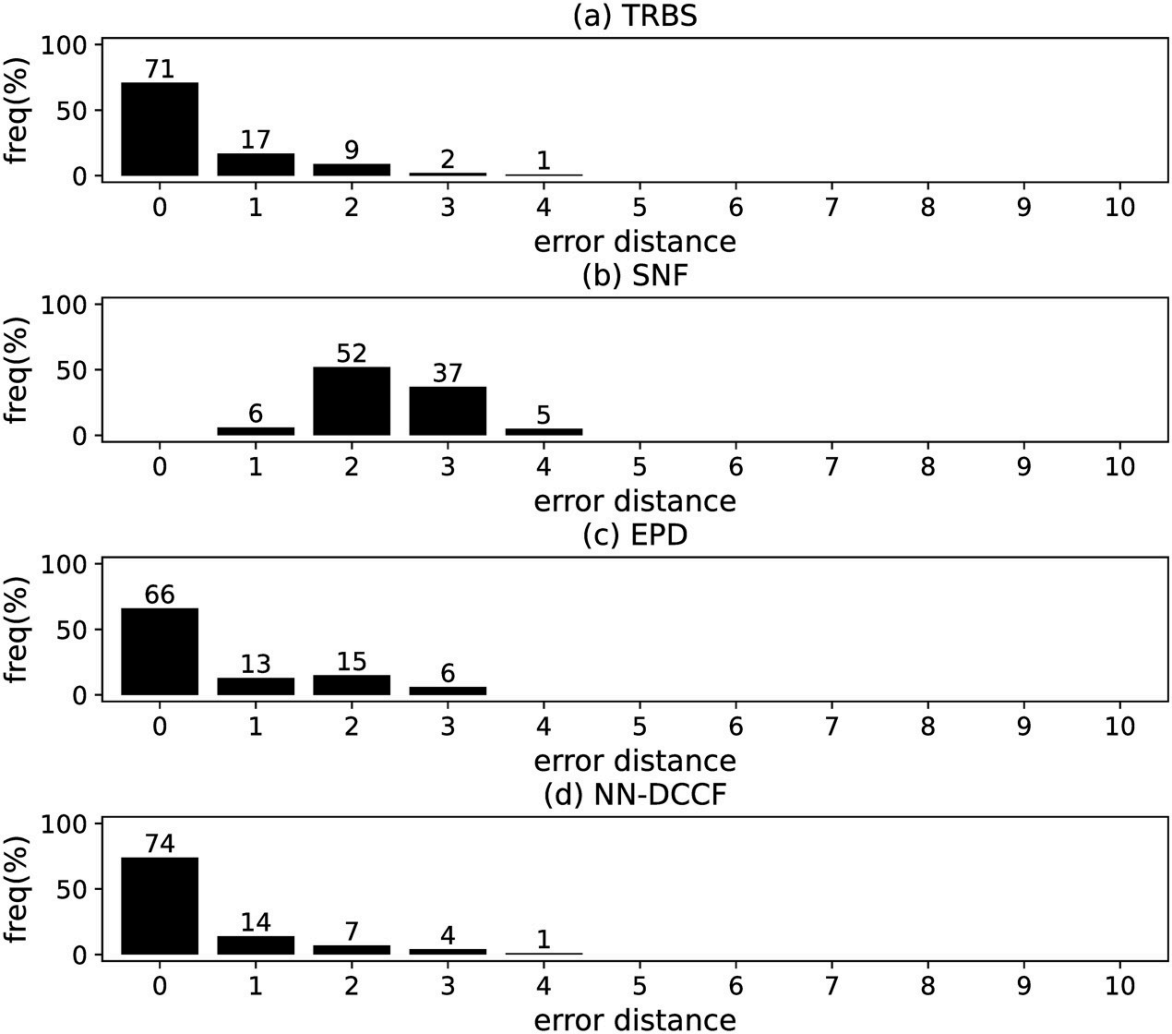
The error distance of TRBS, SNF, EPD and NN-DCCF methods on BA model (1).

**Fig 5:**
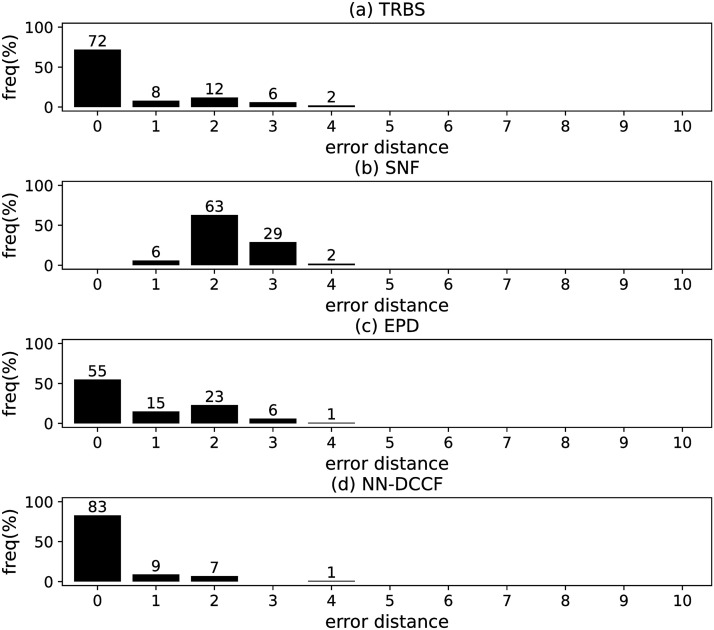
The error distance of TRBS, SNF, EPD and NN-DCCF methods on BA model (2).

**Fig 6:**
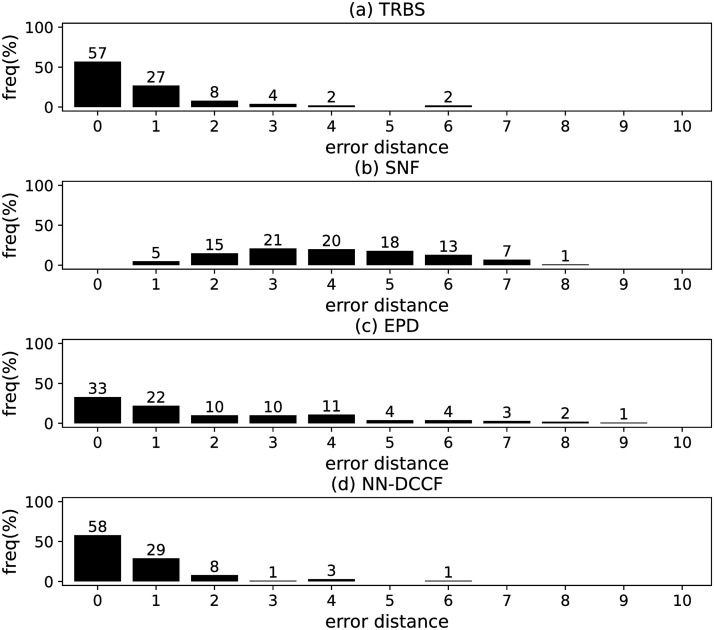
The error distance of TRBS, SNF, EPD and NN-DCCF methods on WS model (1).

**Fig 7:**
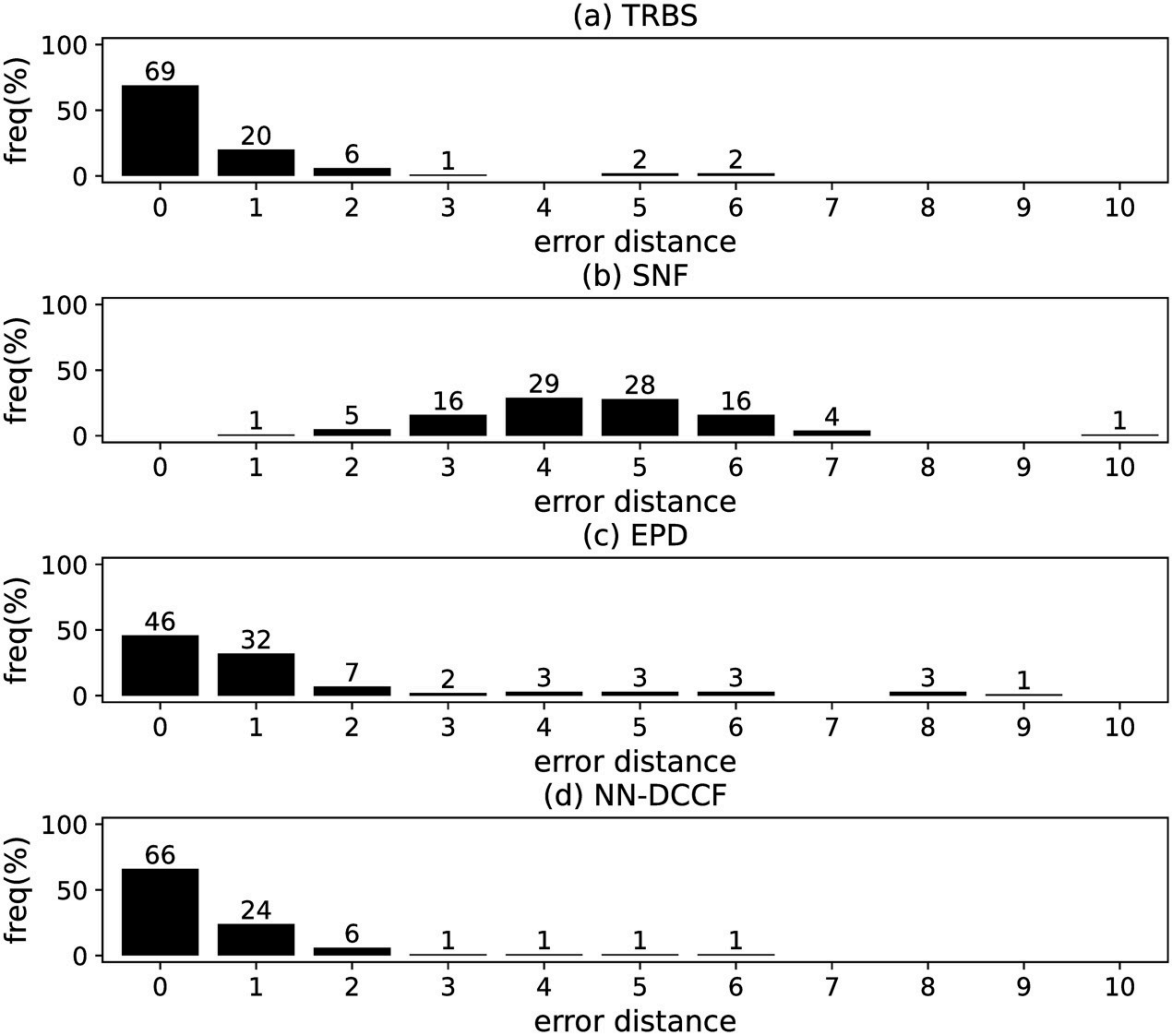
The error distance of TRBS, SNF, EPD and NN-DCCF methods on WS model (2).

**Fig 8:**
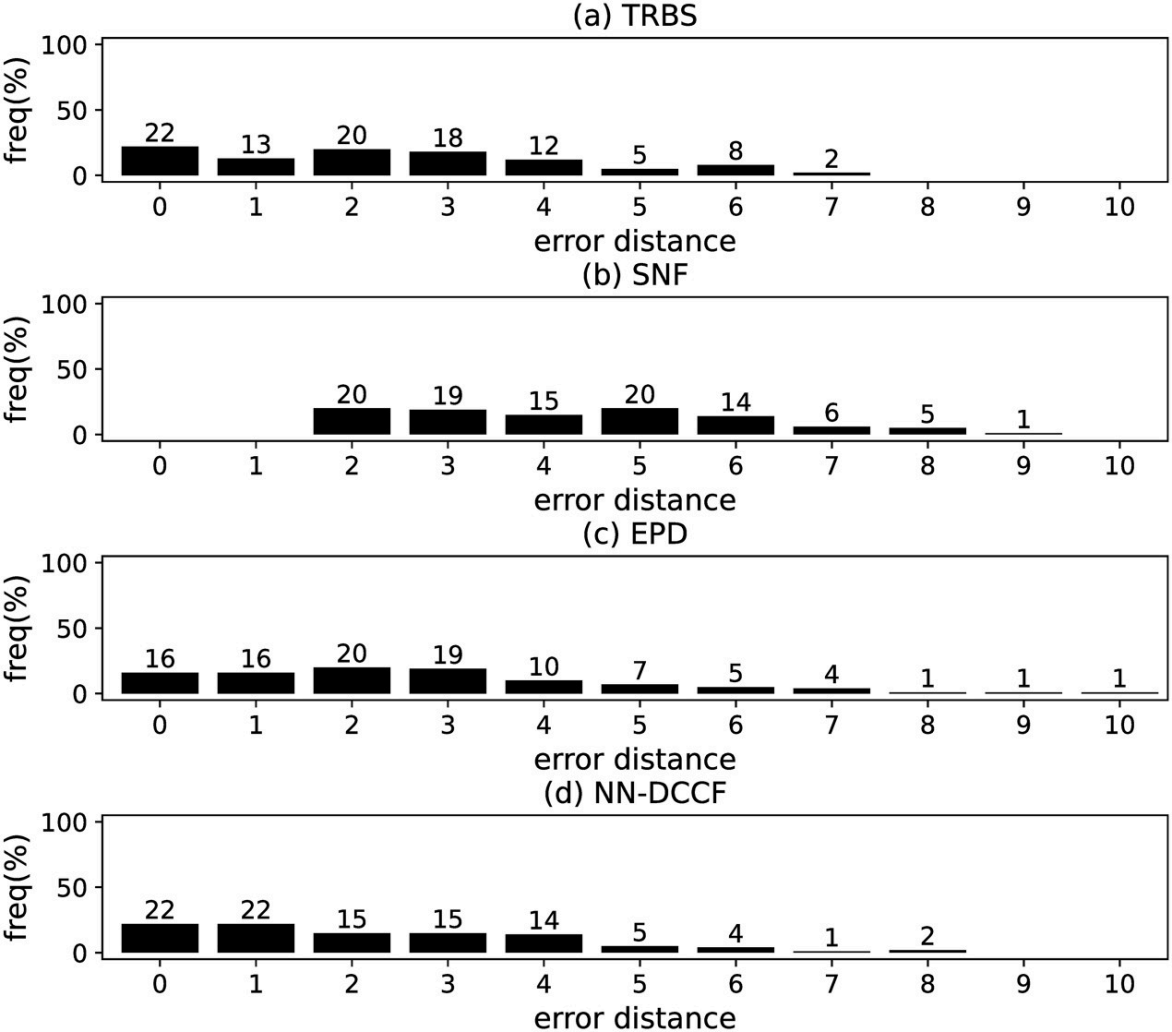
The error distance of TRBS, SNF, EPD and NN-DCCF methods on NetworkScience network.

**Fig 9:**
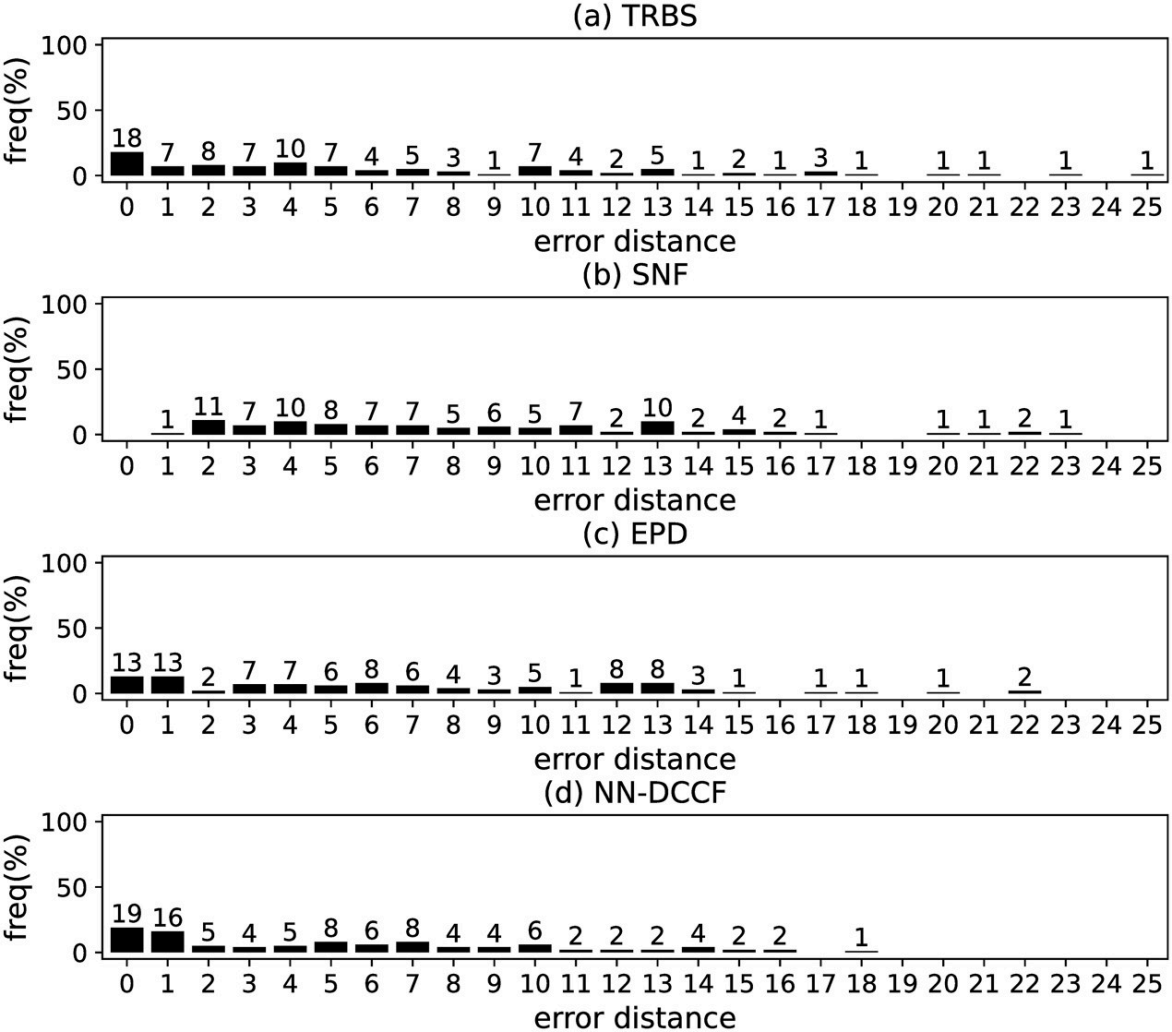
The error distance of TRBS, SNF, EPD and NN-DCCF methods on Euroroads network.

**Fig 10:**
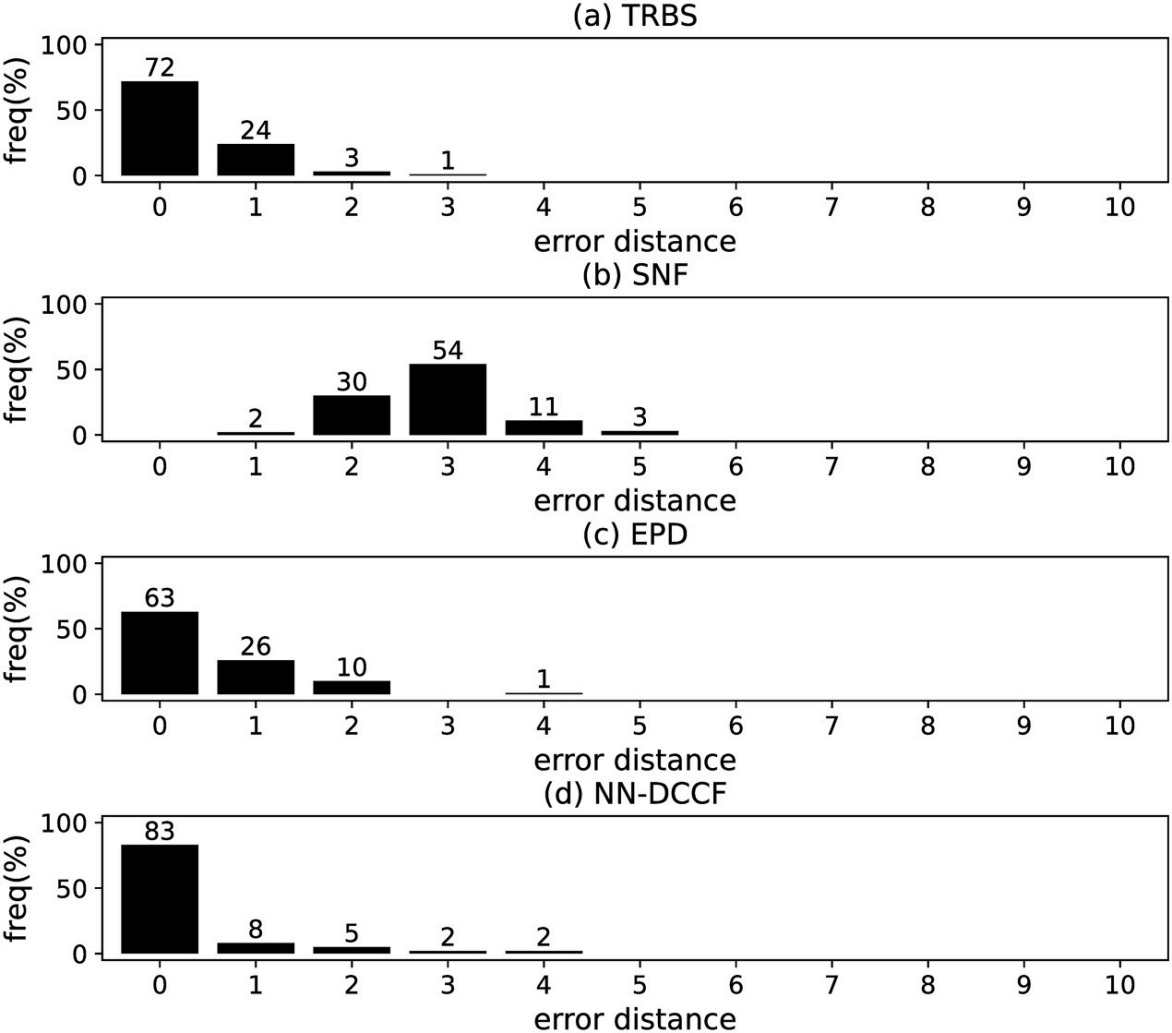
The error distance of TRBS, SNF, EPD and NN-DCCF methods on Email network.

**Fig 11:**
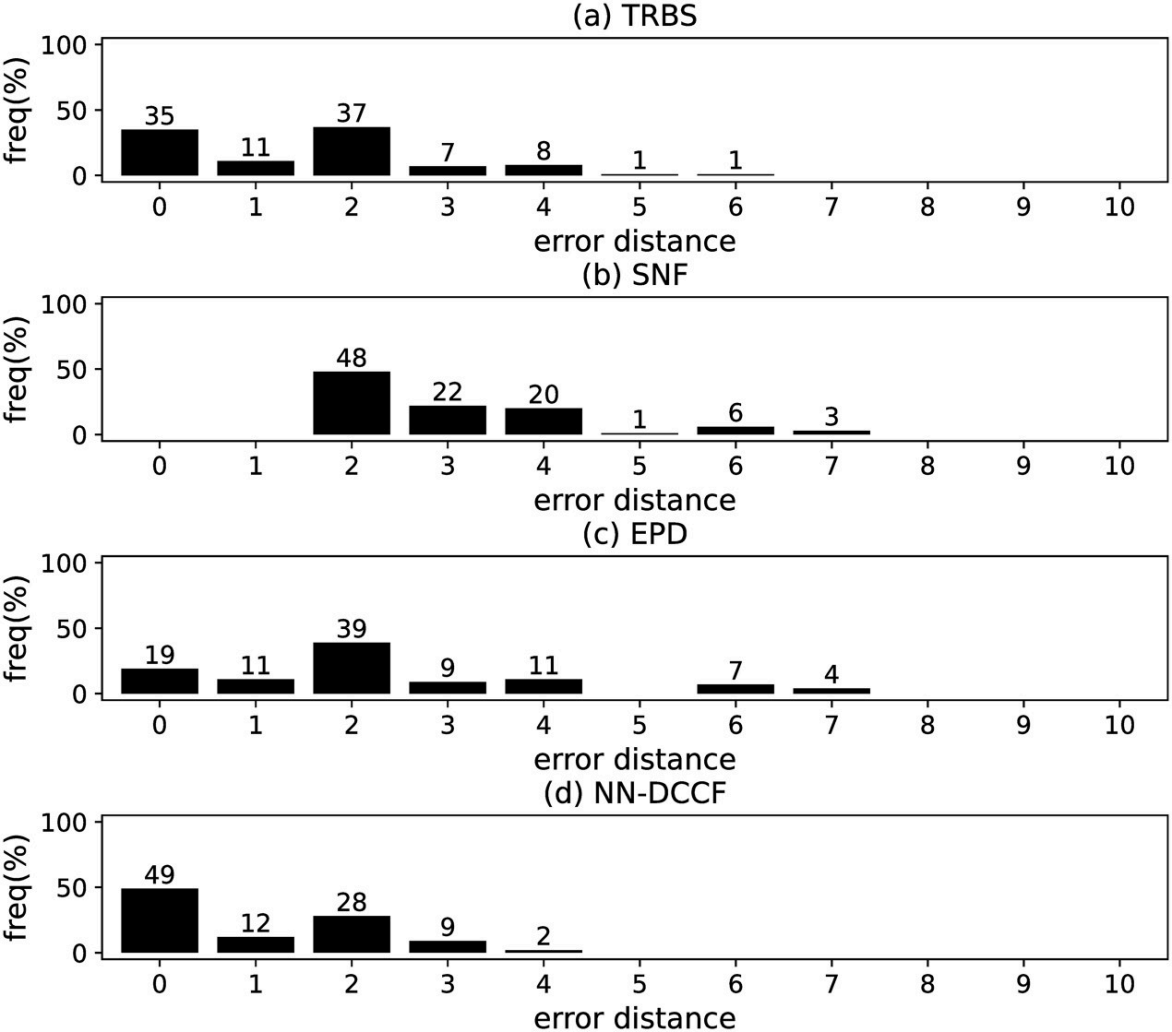
The error distance of TRBS, SNF, EPD and NN-DCCF methods on Blogs network.

The parameters set of GLSTM-AE are summarised in Table 6. Meanwhile, we generate the training dataset of GLSTM-AE for each network with the simple method introduced in Section Stage 2. To emphasize the local structure, we set *l* ∈ [2, 4]. The training dataset size of GLSTM-AE on different networks are shown in Table 7. The training parameters set for GLSTM-AE on different networks are summarised in Table 8. Because the purpose is to identify the diffusion source, we show the accuracy of GLSTM-AE by the results of source identification, which can be found in Figs 4–11 and Table 10.

**Table 6:** The parameters set of GLSTM-AE.

parameter	dimension
*d*_*I*_	|*V*|
*d*_*r*_	8, |*V*|<1000
	16, |*V*|≥1000
*d*_*O*_	|*V*|

*d*_*I*_: input dimension of GLSTM-AE.

*d*_*r*_: the dimension of representation result obtained by GLSTM-AE.

*d*_*O*_: output dimension of GLSTM-AE.

**Table 7:** The training dataset size of GLSTM-AE and SCNN on different networks.

Network	GLSTM-AE	SCNN
BA model (1)	115282	28800
BA model (2)	991382	54000
WS model (1)	21896	28800
WS model (2)	54784	54000
NetworkScience	92902	27288
Euroroads	19752	56106
Email	3714528	101970
Blogs	1253118	143352

**Table 8:** The training parameters set of GLSTM-AE and SCNN on different networks.

Network	GLSTM-AE			SCNN		
	BS	LR	epochs	BS	LR	epochs
BA model (1)	256	0.01	40	256	0.001	20
BA model (2)	1024	0.01	10	1024	0.0001	20
WS model (1)	256	0.03	40	256	0.0002	20
WS model (2)	1024	0.02	40	1024	0.0001	20
NetworkScience	256	0.01	40	256	0.0001	20
Euroroads	1024	0.02	40	512	0.0001	20
Email	2048	0.02	10	2048	0.0002	20
Blogs	2048	0.02	10	2048	0.0002	20

BS: batch size.

LR: learning rate.

The parameters set of SCNN are summarised in Table 9. Further, for each network, we generate the training dataset of SCNN by Algorithm 3. The training dataset size of SCNN on different networks are shown in Table 7. The training parameters set for SCNN are summarised in Table 8. In SCNN, we adopt the batch normalization as the normalization layer [19, 20]. Since the purpose is to identify the diffusion source, we validate the performance of SCNN by the results of source identification, which can be found in Figs 4–11 and Table 10.

**Table 9:** The parameters set of SCNN.

Parameter	dimension
The input of first FC layer	*K* ⋅ *η* ⋅ *l* ⋅ *d*_*r*_
The output of first FC layer	*K* ⋅ *η* ⋅ *d*_*r*_
The input of second FC layer	*K* ⋅ *η* ⋅ *d*_*r*_
The output of second FC layer	|*V*|
LogSoftmax layer	|*V*|

**Table 10:** The average error distance of TRBS, SNF, EPD and NN-DCCF on different networks.

network	TRBS	SNF	EPD	NN-DCCF
BA model(1)	0.45	2.41	0.61	**0.44**
BA model(2)	0.58	2.27	0.83	**0.27**
WS model(1)	0.75	4.03	2.06	**0.66**
WS model(2)	0.57	4.49	1.30	**0.54**
NetworkScience	2.42	4.32	2.73	**2.25**
Euroroads	6.32	8.35	6.62	**5.26**
Email	0.33	2.83	0.50	**0.32**
Blogs	1.49	3.04	2.30	**1.03**

#### Experimental results and discussion

Figs 4–11 show the error distance of the four methods on different networks. Table 10 shows the average error distance of the four methods. From Figs 4 to 11, we can see that the precisions (i.e. the proportion of 0 error hop) exposed by NN-DCCF on the eight networks are 74%, 83%, 58%, 66%, 22%, 19%, 83% and 49%, respectively. Obviously, except for WS model (2), NN-DCCF exposes the best performance in precision. On WS model (2), the precision of NN-DCCF is only inferior to the TRBS, and superior to other two methods. From Table 10, we know that the NN-DCCF is superior to other three methods in the average error distance on all networks. Therefore, the NN-DCCF is a feasible and effective method in accurately identifying the diffusion source. Additionally, from Table 4, we know that the eight networks are different in their topological properties, which indicates that NN-DCCF could effectively identify the source on different types of networks by simply modifying the training parameters. Therefore, the NN-DCCF is a general source identification framework.

## Conclusion

This paper defines the diffusion direction and time information of observers as diffusion characteristics, and develops a NN-DCCF to identify the diffusion source by classifying the diffusion characteristics. Firs, we utilize the diffusion characteristics to construct network snapshot feature. Then, we propose a GLSTM-AE by which the network snapshot feature is represented as low-dimension vectors. Further, we propose a SCNN to identify the diffusion source. By using NN-DCCF, the identification of diffusion source is converted into a classification problem. The feasibility and effectiveness of NN-DCCF are validated by the experimental results on a series of synthetic and real networks. In the future work, we will generalize the NN-DCCF to the case of multi-source.

## Supporting information

S1 File Long short-term memory (LSTM). (PDF)
