# Supplementary material for: Diffusion characteristics classification framework for identification of diffusion source in complex networks
Source: PLoS One. 2023 May 15;18(5):e0285563. doi: 10.1371/journal.pone.0285563 (PMC10184948; doi:10.1371/journal.pone.0285563)
Supplement: S1 File — (PDF) [file pone.0285563.s001.pdf]

Supporting information

Long short-term memory (LSTM). Long short-term memory (LSTM) [1] is an improved variant of recurrent neural networks (RNN). LSTM develops a memory cell to store information and incorporates several control gates to read and write the memory cell, which has shown superior performance in learning the dependence information of long and short time series. Currently, LSTM has been extensively used to perform various sequence learning tasks [2-5]. The LSTM computes a mapping from an input sequence to an output sequence by using the following update equations:

$$i_t = \sigma\left(W_{ii}x_t + b_{ii} + W_{hi}h_{t-1} + b_{hi}\right) \tag{3}$$

$$f_t = \sigma\left(W_{if}x_t + b_{if} + W_{hf}h_{t-1} + b_{hf}\right) \tag{4}$$

$$g_t = \tanh\left(W_{ig}x_t + b_{ig} + W_{hg}h_{t-1} + b_{hg}\right) \tag{5}$$

$$o_t = \sigma\left(W_{io}x_t + b_{io} + W_{ho}h_{t-1} + b_{ho}\right) \tag{6}$$

$$c_t = f_t \odot c_{t-1} + i_t \odot g_t \tag{7}$$

$$h_t = o_t \odot \tanh\left(c_t\right) \tag{8}$$

where, the W terms denote weight matrices, the b terms denote bias vectors. σ is the sigmoid function. $\odot$ denotes element-wise product. h_t is the hidden state at time t. c_t is the cell state at time t. x_t is the input at time t. h_{t-1} is the hidden state of the layer at time $t-1$ or the initial hidden state. i_t, f_t, g_t and o_t are the input, forget, cell and output gates, respectively.

References

1. Hochreiter S, Schmidhuber J. Long short-term memory. Neural computation. 1997; 9(8): 1735–1780. https://doi.org/10.1162/neco.1997.9.8.1735

2. Sutskever I, Vinyals O, Le QV. Sequence to Sequence Learning with Neural Networks. Advances in Neural Information Processing Systems. 2014; 27. Available: https://proceedings.neurips.cc/paper/2014/file/a14ac55a4f27472c5d894ec1c3c743d2-Paper.pdf

3. Srivastava N, Mansimov E, Salakhudinov R. Unsupervised learning of video representations using lstms. International conference on machine learning. 2015. pp. 843–852. Available: http://proceedings.mlr.press/v37/srivastava15.pdf

4. Zhang C, Zhao S, He Y. An Integrated Method of the Future Capacity and RUL Prediction for Lithium-Ion Battery Pack. IEEE Transactions on Vehicular Technology. 2022; 71(3): 2601–2613. https://doi.org/10.1109/TVT.2021.3138959

5. Zhao S, Zhang C, Wang Y. Lithium-ion battery capacity and remaining useful life prediction using board learning system and long short-term memory neural network. Journal of Energy Storage. 2022; 52: 104901. https://doi.org/10.1016/j.est.2022.104901
